# On the Injection of Urea and Other Substances into the Blood

**Published:** 1858-05

**Authors:** William A. Hammond

**Affiliations:** Assistant Surgeon U. S. Army


*On the Injection of Urea and other substances into the blood.* By WILLIAM A. HAMMOND, M. D., Assistant Surgeon U. S. Army. Read before the Biological Society of Philadelphia, January 18th, 1858.

The principal object in view in undertaking the experiments detailed in this paper, was that of deciding upon the correctness of the theory advanced by Frerichs,* explanatory of uræmic intoxication. As is well known, this distinguished author regards the symptoms of blood poisoning, so frequently present in Bright's disease, as not directly depending upon the presence of urea in this fluid, but as being caused by its conversion, through the agency of a ferment, into carbonate of ammonia.

Frerichs performed two series of experiments, which he regards as tending to sustain his hypothesis. In the first series he injected a solution of urea into the blood of animals whose kidneys had been previously removed. In from 1¼ to 8 hours they became restless and vomited.—Ammonia was detected in the expired air, and simultaneously convulsions ensued. Death occurred in from 2½ to 10 hours from the commencement of the experiment. Ammonia was found in the blood, the contents of the stomach, and in the bile and other secretions.

In the second series a solution of carbonate of ammonia was injected. Convulsions ensued almost immediately, and were quickly followed by stupor. The respiration was labored, and the expired air was loaded with ammonia. This substance, however, gradually disappeared, and the animals recovered their senses.

Frerichs offers no explanation of the nature of the ferment which he conceives to be necessary to produce uræmic poisoning, nor does he even attempt to demonstrate its existence, except indirectly, through the experiments above cited.

While admitting the facts set forth by these experiments, I am constrained to differ with Frerichs in his theory. Ammonia has often been

* Die Bright'sche Fierenkrankheit und deren Behandlung.

met with as a constituent of the expired air of healthy individuals. I have myself frequently detected it in such cases; it has been demonstrated to be constantly present in the blood; and Frerichs' own experiments (those of the second series) show that it was not capable of causing death even when injected directly into the circulation, and when its presence in the blood was evidenced by its being exhaled in large quantity from the lungs.

The fact that in the first series of investigations the kidneys were extirpated, while in the second the animals unmutilated, while different substances were used in each, prevents our drawing any comparative conclusions from the results obtained.

The experiments to which the present paper relates consisted of two series. In the first the substances were injected into the blood of the sound animal; in the second the kidneys were previously extirpated.—The two series were, as far as possible, alike in every other respect. The substances injected in both series were urea, urea and vesical mucus, carbonate of ammonia, nitrate of potash, and sulphate of soda.

FIRST SERIES.

1*st Experiment.* UREA.—Into the jugular vein of a large dog I carefully injected 60 grains of urea, dissolved in 4 ounces of distilled water.

No immediate effect was produced. After the lapse of 15 minutes the respiration became more hurried, and the animal began to show signs of uneasiness. At the end of 1½ hours slight spasms of the limbs ensued, and lasted about 10 minutes. These were followed by a disturbed sleep of two hours' duration. The dog then awoke, passed a large quantity of urine, and seemed perfectly well; no other abnormal symptoms occurred.

Ammonia was at no time detected on holding a rod, previously dipped in chlorohydric acid, in the current of the expired air.

The urine of this dog, for the twenty-four hours immediately preceding the commencement of the experiment, amounted to 932 cubic centimetres, and contained 287·39 grains of urea. For the twenty-four hours commencing with the experiment, the quantity of urine was 1268 cubic centimetres, and the urea 341·12 grains—a difference of 336 cubic centimetres of urine and 53·73 grains of urea in favor of the second day. The solid food on both days was the same; the amount of water drank was less on the second day than the first.

From this experiment it is perceived that, of the 60 grains of urea injected into the circulation, 53 73 grains were recovered from the urine, leaving a balance of but 6·27 grains unaccounted for; but which, however, was probably excreted with the urine under some othor form.

2*d* *Experiment.* Urea and Vesical Mucus.—60 grains of urea were dissolved in 5 ounces of distilled water, mixed with 115 grains of vesical mucus,* and carefully introduced into the jugular vein of a dog. The symptoms which followed were almost identical with those of the first experiment, except that the sleep was about 30 minutes longer. On awaking a large quantity of urine was passed. No Ammonia was discovered in the breath. The animal recovered perfectly.

For the twenty-four hours previous to this experiment, the urine of this dog amounted to 823 cubic centimetres, and contained 194·21 grains of urea. For the second period of the twenty-four hours the urine amounted to 1459 cubic centimetres, and the urea to 272·86 grains, being an increase of 536 cubic centimetres of urine and 78·65 grains of urea—18·65 grains more than were injected into the circulation. The solid food was the same on both days; the water somewhat more on the second than the first.

3*d* *Experiment.* Carbonate of Ammonia.—60 grains of carbonate of ammonia, dissolved in 4 ounces of water, were injected into the jugular vein of a large dog. Symptoms of great uneasiness almost immediately followed. The animal staggered about the room, and, after a few moments, fell and lay panting and moaning on the floor. At the end of two minutes, copious fumes of chloride of ammonium were produced by holding a rod, on which were a few drops of chlorohydic acid, to the mouth. Convulsions ensued at the end of 5½ minutes from the commencement of the experiment, and continued 10 minutes. They than ceased, and did not recur. Ammonia continued to be evolved from the lungs for 1¾ hours. The dog remained in the recumbent posture for two hours. Three hours after the commencement of the experiment, he evacuated a quantity of ammoniacal urine. He recovered perfectly.

During the twenty-four hours preceding this experiment, the dog eliminated 1327 cubic centimetres of urine, which contained 345·82 grains of urea. For the succeeding period the quantity of urine was 1654 cubic centimetres, and the amount of urea 296·53 grains, an increase of 227 cubic centimetres in the urine, and a diminution of 48·29 grains in the urea. The food was of the same character and quantity on both days; the amount of water drank was slightly greater on the second day.

The symptoms observed after the introduction of the carbonate of ammonia into the blood, it is seen, did not correspond altogether with those

---

* It is well known that to this latter substance is ascribed the power of converting urea into carbonate of ammonia out of the system. I was desirous of ascertaining how far its influence could be exerted in the blood within the system.

which followed in Frerich's investigations. Thus, there was no vomiting nor stupor, and the convulsions were not of so violent a character. The quantity of carbonate of ammonia injected by Frerich is not stated by him, and the difference in the effects may be due to a difference in the amount of this substance introduced into the circulation.

*4th Experiment.* NITRATE OF POTASH.—I injected 60 grains of nitrate of potash, dissolved in 4 ounces of water, into the circulation of a medium sized dog. Convulsions of a violent character almost instantly ensued, and continued, with occasional intermissions, for 1¾ hours. There was also vomiting and severe retching. Stupor followed, and lasted till the death of the animal, which took place at the end of 5 hours and 25 minutes from the commencement of the experiment. No ammonia was detected in the breath.

*5th Experiment.* SULPHATE OF SODA.—Sixty grains of sulphate of soda, dissolved in four ounces of water, were injected into the jugular vein of a medium sized dog. Convulsions quickly followed, and were very severe, lasting about two and a half hours, with short intermissions. The dog vomited twice. Stupor ensued upon the convulsions, and was present for about three hours. The dog gradually recovered his senses, but was disposed to remain quiet fer the balance of the day. The next day he seemed entirely well.

The urine of the twenty-four hours preceding the experiment amounted to 1125 cubic centimetres, and the urea to 211·34 grains. For the twenty-four hours commencing with the experiment, the quantity of urine was 1283 cubic centimetres, and of urea 201·15 grains—an increase in the urine of 158 cubic centimetres, and a decrease in the urea of 6·19 grains.

### SECOND SERIES.

In this series, as before remarked, the kidneys were removed previously to the introduction of the substances experimented with into the circulation. Extirpation of these organs is not necessarily attended with an immediately fatal result. A dog will live from one to four days after this operation, when it has been carefully performed.

*1st Experiment*, UREA.—I removed the kidneys from the dog used in the first experiment of the foregoing series. He bore the operation exceedingly well, and even appeared somewhat lively after it. Three hours subsequently I injected 60 grains of urea, dissolved in 4 ounces of water, into the jugular vein. Convulsions ensued at the end of 45 minutes, and continued with alternations of stupor for 6¼ hours, when the animal died. There was no vomiting. No ammonia was at any time detected in the breath.

The post-mortem examination showed a healthy condition of the stomach and bowels. No ammoniacal odor was perceived.

A portion of the fluid contents of the stomach were mixed with a little caustic baryta, in a test tube, and gently heated. On holding a glass rod, moistened with chlorohydric acid, over the mouth of the tube, no fumes of chloride of ammonium were formed, showing, therefore, the absence of ammonia.

By employing caustic potash instead of baryta, and then applying the glass rod as before, copious fumes of the chloride of ammonium were produced. This arose from the conversion of the urea present into carbonate of ammonia through the action of the potash.

The presence of urea in the stomach was directly determined by evaporating a portion of the fluid contents to dryness, at a low heat, by means of the water-bath, treating the residue with alcohol, and again evaporating to dryness. By digesting the solid residue with warm water, filtering, and adding nitric acid to the filtrate, crystals of nitrate of urea were formed in considerable quantity.

The fact that no ammonia was discovered in the breath or contents of the stomach of this animal is in direct opposition to the results of Frerich's experiments. I think it will be generally admitted that had any ammonia been present, it would have been detected by the means employed, and conjoined to the fact that such unequivocal evidences of the existence of urea in the stomach were indicated, leave no doubt of the non-conversion in this instance, at least, of the urea into carbonate of ammonia.

The death in this case is, therefore, I think, fairly to be attributed to the direct action of the urea upon an organism brought into an abnormal condition by removal of the kidneys. In Bright's disease a similar condition of the system is present, and may exercise a like influence over the action of the urea which has accumnlated in the blood.

*2d Experiment.* Urea and Vesical Mucus.—Having previously extirpated the kidneys from the dog used in the corresponding experiment of the foregoing series, I injected into the circulation 60 grains of urea, dissolved in 4 ounces of water, to which 115 grains of vesical mucus had been added. The dog remained quiet for nearly an hour, when violent convulsions ensued, and continued, with but slight intermissions, for 5½ hours. Vomiting of billious matters occurred but twice. Stupor followed the convulsions, and remained till death, which took place at the end of 8 hours and 20 minutes from the commencement of the experiment.

No ammonia was detected in the expired air or vomited matters, nor was any discovered after death in the contents of the stomach. Urea was indicated in these latter by the method employed in the previous experiment.

The same remarks are applicable to this experiment as to the preceding, as the results are almost identical. In addition, it will be remarked that the mucus injected exercised no influence on the composition of the urea introduced into the circulation with it.

3*d Experiment.* Carbonate of Ammonia.—After extirpating the kidneys, I injected into the jugular vein of the dog previously used in the corresponding experiment of the preceding series, 60 grains of carbonate of ammonia, dissolved in 4 ounces of water. Convulsions ensued in five minutes, and continued almost uninterruptedly for 3½ hours. Stupor followed, and remained present till the death of the animal, which took place 6 hours and 18 minutes from the commencement of the experiment. During the convulsions there were several times vomiting of chyme and mucus which gave a strong ammoniacal odor.

Ammonia was detected in the breath in 2½ minutes after the injection into the blood. No post-mortem examination was made.

From this experiment it is seen that carbonate of ammonia is speedily fatal after being introduced into the circulation of an animal deprived of its kidneys.

4*th Experiment.* Nitrate of Potash.—Sixty grains of nitrate of potash were introduced, dissolved in 4 ounces of water, into the circulation of a medium sized dog whose kidneys had been removed three hours before. Convulsions followed in 4½ minutes, and continued for 3½ hours, when the animal died. There was neither vomiting nor stupor, but undoubted evidence of the existence of ammonia in the breath was obtained. The post-mortem examination showed a compacted condition of the lungs and of the vessels of the stomach.

5*th Experiment.* Sulphate of Soda.—I removed the kidneys from the dog used before in the corresponding experiment, and 3 hours afterward injected 60 grains of sulphate of soda, dissolved in 4 ounces of water, into his circulation. Convulsions came on in 3 hours and 20 minutes, and lasted about two hours, when stupor supervened, and the animal quickly died. There was no vomiting. No post-mortem examination was made.

Judging from the foregoing experiments, I think that Frerich's theory of uræmic intoxication is erroneous. In neither of the cases in which urea was injected into the circulation was any ammonia detected in the breath, vomited matters, or contents of the stomachs of the animals. Without pretending to question the accuracy of Frerich's statement relative to the discovery of ammonia in his experiments where urea was injected, I am of opinion that its presence was accidental, and that it is not to be regarded as an invariable attendant upon the retention of urea in the system.

Removal of the kidneys would seem to exercise a very important influence over the action of substances which, when introduced directly into the blood of sound animals, are not capable of causing death, or even of producing much constitutional disturbance. Thus, of ten animals experimented upon in the first series, but one (that in which nitrate of potash was injected) died, while all of the second series were attacked with convulsions and sank after a few hours. It is seen, therefore, that carbonate of ammonia is not more poisonous than the other substances used, and not so much so as nitrate of potash.

The condition of system remaining after extirpation of the kidneys is, in many respects, analagous to that present during Bright's disease. In the latter condition the kidneys act imperfectly, and many substances which should be eliminated are retained in the organism. In the former there is, of course, no excretion of matter through these channels. In all cases, so far as I am aware, where the kidneys of animals have been extirpated and urea injected into the blood, death has supervened in a much shorter time than would have been the case had no urea been thus introduced into the system. I see, therefore, no great difficulty in ascribing the condition known as uremic intoxication to the direct action of an excessive accumulation of urea in the system.

The fact that urea in large quantity has been found in the blood of persons suffering under Bright's disease, but in whom no symptoms of blood poisoning were present, is no argument against the correctness of this view, for, doubtless, as with most other poisons, all persons are not alike sensitive to its action. Moreover, when the disease progresses slowly, the rate of accumulation of urea is also slow, and thus the system by becoming in a manner habituated to its presence, may be enabled to endure an excess without symptoms of intoxication necessarily attending.

I conclude, therefore, from the foregoing experiments—

1st. That urea, (simple and combined with vesical mucus,) carbonate of ammonia, and sulphate of potash, when injected into the blood-vessels of sound animals, do not cause death.

2d. That nitrate of potash, when thus introduced, is speedily fatal.

3d. That death ensues from the injection of any of the foregoing named substances into the circulation of animals whose kidneys have been previously extirpated.

4th. That in neither case does urea, when introduced directly into the circulation, undergo conversion into carbonate of ammonia.

[*North American Medico-Chirurgical Review.*

